# Implementation of cross-sectoral rehabilitation in the Nordic countries: a scoping review

**DOI:** 10.3389/frhs.2025.1662230

**Published:** 2025-09-19

**Authors:** Helle Bøgard, Signe Green Knakkergaard, Charlotte Simonÿ, Lars Hermann Tang, Jeanette Reffstrup Christensen, Anne Dalhoff Pedersen, Alexander Luijk, Stine Gundtoft Roikjær

**Affiliations:** ^1^ The Research and Implementation Unit PROgrez, Department of Physiotherapy and Occupational Therapy, Næstved-Slagelse-Ringsted Hospitals, Slagelse, Denmark; ^2^ The Department of Regional Health Research, University of Southern Denmark, Odense, Denmark; ^3^ Research Unit of General Practice, Department of Public Health, University of Southern Denmark, Odense, Denmark; ^4^ Research Unit for General Practice, Aarhus, Denmark; ^5^ DRIVEN – Danish Centre for Motivational and Behavior Science, Department of Sports Science and Clinical Biomechanics, University of Southern Denmark, Odense, Denmark; ^6^ Centre for Health Research, Zealand University Hospital, Nykøbing Falster, Denmark; ^7^ Center for Health, University College Absalon, Roskilde, Denmark

**Keywords:** cross-sectoral, implementation, rehabilitation, Nordic welfare systems, organization

## Abstract

**Introduction:**

Rehabilitation needs are rising in the Nordic countries due to an aging population and declining health profiles**.** Nordic healthcare systems share common features, including universal access, organization, and substantial tax-based financing. Due to the organization of the healthcare system, patients often experience transitions between sectors as part of the rehabilitation program. This fragmented setup undermines the continuity and quality of rehabilitation, making implementation more difficult. To inform future implementation processes, this scoping review examines the factors that influence cross-sectoral rehabilitation in settings with comparable healthcare systems.

**Methods:**

This Scoping review followed the Preferred Reporting Items for Systematic Reviews and Meta-Analyses Extension for Scoping Reviews. The search strategy aimed to identify published, peer-reviewed primary studies on interventions implemented in adult rehabilitation within Nordic countries. Data were charted following Levac et al.'s framework and analyzed using Elo & Kyngäs' content analysis to identify factors influencing implementation. Key study characteristics and implementation approaches were synthesized narratively and in tables.

**Results:**

Thirty-six papers were identified. Most studies described the implementation of rehabilitation transitioning from the secondary to the primary sector. A top-down implementation approach was predominantly reported and appears more facilitating than a bottom-up approach. Implementation of rehabilitation across sectors is influenced by an interplay of factors: (1) Organization & Resources: alignment of context with intervention, involvement from front-line personnel, time & resources, the workplace itself, and managers, and (2) Collaboration & Communication, including knowledge and competence, attitudes, communication, patients, and families.

**Conclusion:**

While this scoping review conveys that collaboration, communication, resources, and organization have a central role affecting the implementation of cross-sectoral rehabilitation, it further identifies knowledge gaps, such as the lack of the patients' perspective, the use of a framework or other systematic approach to ensure the success of the implementation.

## Introduction

The population in the Nordic countries is aging rapidly (1), with over 20% now aged 65 years and older (2). Accompanying this increasing age brings a growing burden of chronic -and mental conditions, along with functional limitations. These issues often require complex journeys across multiple healthcare sectors (3). In this process, rehabilitation services become critical. They support individuals in regaining function, independence, and quality of life (4–6).

Nordic healthcare systems share features such as universal access, decentralization, and substantial tax-based financing. These systems cover the majority of healthcare expenditures (3). The healthcare services in Nordic countries are organized across primary, secondary, and sometimes, tertiary sectors. This structure leads to patient transitions between sectors and settings during rehabilitation (3). These transitions have challenged the continuity and quality of rehabilitation (7).

Current implementation research in Nordic countries and other comparable healthcare contexts has often focused on specific areas. These include transitional care for older adults (7), improvements in primary care (8) and mental health interventions (9, 10). Some studies have also focused the roles of particular professional groups, such as nurses (11). Although valuable, these studies do not fully capture the complexity of implementing cross-sectoral rehabilitation for the general adult population. A notable gap remains in understanding how to implement interventions that span multiple organizations. Such efforts often require collaboration among diverse professional groups across sectors. Implementation science recognizes that successfully integrating interventions into real-world settings is an iterative and dynamic process. It is also influenced by a range of ever-changing determinants (12). Defining and understanding these determinants is challenging, especially when interventions are not confined to a single context or profession. Successful cross-sectoral implementation may depend upon various fundamental components. These include stakeholder engagement, alignment of organizational missions, and use of an implementation framework. Interprofessional collaboration, and cultural adaptability within and across different healthcare settings are also essential (13–17). Contextual factors, such as institutional structures, resource availability, and normative values, can significantly shape the uptake, sustainability, and scalability of rehabilitation (7, 18, 19).

To advance implementation, it is crucial to investigate how these factors converge and influence cross-sectoral rehabilitation in settings where healthcare systems, financing models, and sociocultural values are relatively comparable (1, 2, 20). By focusing on the Nordic countries, we can explore processes and outcomes within similar welfare-based health systems and populations. This shared context provides a more controlled environment to identify factors that either facilitate or hinder successful implementations. Such insights can guide the design, adaptation, and evaluation of future interventions, ultimately optimizing the delivery of cross-sectoral rehabilitation services.

In light of these considerations, our scoping review aims to illuminate the factors, including both facilitators and barriers, that influence the implementation of cross-sectoral rehabilitation in Nordic healthcare settings. By synthesizing the available evidence, we seek to address current knowledge gaps, improve clarity on implementation strategies, and stimulate further development of context-specific implementation strategies. This endeavor enriches our understanding within the Nordic context. It also contributes broadly to the field of implementation science, offering transferable insights. These insights can support effective translation of evidence-based interventions across diverse healthcare systems and populations.

### Aim

This scoping review aims to systematically examine and synthesize existing research on the cross-sectoral implementation of rehabilitation in Nordic health systems, focusing on factors that influence implementation processes to inform and support future implementation efforts.

## Methods

A scoping review was chosen to examine the extent, nature, and range of existing peer-reviewed research literature on the implementation of rehabilitation (21). The review was conducted in accordance with the Preferred Reporting Items for Systematic Reviews and Meta- Analyses Extension for Scoping Reviews (PRISMA-ScR) (22). HB and SGR developed an initial study protocol (https://doi.org/10.17605/OSF.IO/XF6G2) to address the review objectives. A preliminary search was carried out to assess the available literature and refine the search strategy, aligning with the iterative process of the scoping review, which allows for revisiting earlier stages in response to unexpected findings and emerging insights. The scoping review study is not subject to ethical restrictions.

### Eligibility criteria

To be eligible for inclusion in this review, the studies had to:

• Target an adult population aged 18 years or older
• Be conducted within a rehabilitative context
• Report on the implementation of an intervention
• Be located in a Nordic country

Rehabilitative intervention: The World Health Organization (WHO) (23) defines a rehabilitative intervention as “a set of interventions designed to optimize functioning and reduce disability in individuals with health conditions in interaction with their environment”. These interventions were focused on care, physical, mental, and/or social outcomes and were either directed towards patients or aimed at the development and support of healthcare professionals or organizations (23).

#### Implementation

The included studies must evaluate the process and outcomes of implementation. Implementation was defined as the adoption and integration of evidence-based health interventions into routine practice within specific settings (24). This implementation of a rehabilitative intervention must occur in a cross-sectoral setting, meaning it was implemented across or within multiple sectors of the health system. The health system encompasses both public and private entities, including hospitals, municipalities, clinics, general practitioners, nursing homes, and rehabilitation centers.

Studies were excluded if they were: protocols, reviews, opinion pieces, editorials, conference proceedings, or published only as an abstract.

### Data sources

The following databases were searched to identify relevant studies: MEDLINE (via OVID), EMBASE (via OVID), CINAHL (via EBSCOhost), Physiotherapy Evidence Database (PEDro), Occupational Therapy Systematic Evaluation of Evidence (OTseeker), Klinisk Sygepleje (via Idunn) and Web of Science. No limitations or restrictions were applied regarding language or date of publication. All databases were systematically searched in November 2023. The final search strategies can be found in Supplementary File S1. In addition, the full-text articles were hand-screened for additional records as well as run through Web of Science, adding a snowballing effect to the database search. All studies identified through the search strategy were uploaded into Covidence for study selection. Here, all duplicates were excluded.

### Search strategy

The search strategy aimed to identify published, peer-reviewed primary studies. Grey literature was excluded to keep the scope of the review manageable and to ensure a transparent and reproducible search process. An initial search in Cochrane was conducted to expand the search and index terms. These keywords and index terms were tailored to each information source. Two information specialists peer-reviewed the search strategy in accordance with the PRESS guidelines (25).

A four-part PICO model consisting of Population, Intervention, Context/Setting, and Outcome (26) inspired and guided our search. For each component of the PICO model, a combination of Medical Subject Headings (MeSH), keywords, free-text terms, and word variants was employed to ensure comprehensive coverage. For the Population component, the focus was on adults aged 18 years and older residing in the Nordic countries, with country-specific identifiers such as *Denmark, Danish, Sweden, Swedish, Norway, Norwegian, Finland, Finnish, Iceland, Icelandic*. The Intervention component targeted rehabilitation-related concepts, including terms such as *rehabilitation, exercise, physical activity, activities of daily living, leisure activities, recreational therapy*. For the Context/Setting, the emphasis was on cross-sectoral care and transitional processes, with terms such as *cross-sector, hospital discharge, continuity of patient care*. Finally, the Outcome component focused on implementation processes and evaluations, with terms including *implementation, outcome assessment, program development, quality control, delivery of health.* This comprehensive search strategy was designed to capture relevant literature addressing the implementation and evaluation of rehabilitation interventions across sectors in Nordic adult populations (Supplementary File S1).

### Study selection

To enhance consistency among reviewers, each reviewer initially screened 20 papers to become familiar with the inclusion and exclusion criteria and then discussed any questions or concerns regarding the screening process with HB. Following this, the inclusion and exclusion criteria were refined, particularly regarding the definitions of rehabilitative interventions and implementation evaluations. The exclusion criteria encompassed protocols, opinion papers, conference proceedings or similar, abstract-only and reviews and palliative rehabilitation articles. At least two reviewers independently (HB, AL, IU, TGH, SGK, SFH) screened titles and abstracts for eligible studies. Any disagreements were resolved by consensus between the two reviewers. If consensus could not be achieved, a third reviewer was consulted. Studies that passed the initial screening were then reviewed in full text by two reviewers (HB, SGK), who independently decided whether the study should be included. Disagreements were resolved by consensus between the two reviewers. Decisions were discussed and resolved with SGR if consensus could not be achieved.

### Data extraction

The authors followed Levac et al.'s (26) recommendations for advancing scoping methodology to chart the data. A data charting form was developed to extract data from the studies included (Supplementary File S2). The items selected for extraction were chosen to give a thorough insight into each study on design, characteristics, and results and enable a basis for comparison between studies. Items for extraction are country of origin, year of publication, aim, study participants, sectors included, setting, methods, main conclusion, framework, top-down or bottom-up approach, intervention description and the implementers. Two reviewers (HB, SGK) independently charted the data, and then each reviewer verified the other's charting. Disagreements were discussed, and if consensus could not be reached, a third reviewer (SGR) was consulted. The charting process was iterative, and when it became evident that the organizational approach was an element of importance, an additional column with “Top-down, Bottom-up” was added. “Top-down” or “Bottom-up” referred to the implementation process' origin and lead, as to who decided and initiated the implementation. A top-down implementation is decided upon and administered from the top of an organization, for example a national cancer reform (27). A bottom-up implementation is driven and co-designed by the front line healthcare e.g., a patient trajectory where the healthcare professionals define challenges and propose solutions (28).

The charted characteristics are synthesized and presented narratively and in tables. All decisions were discussed between HB and SGK, and if there were any disagreements, SGR was consulted.

### Data analysis

**Figure 1:**
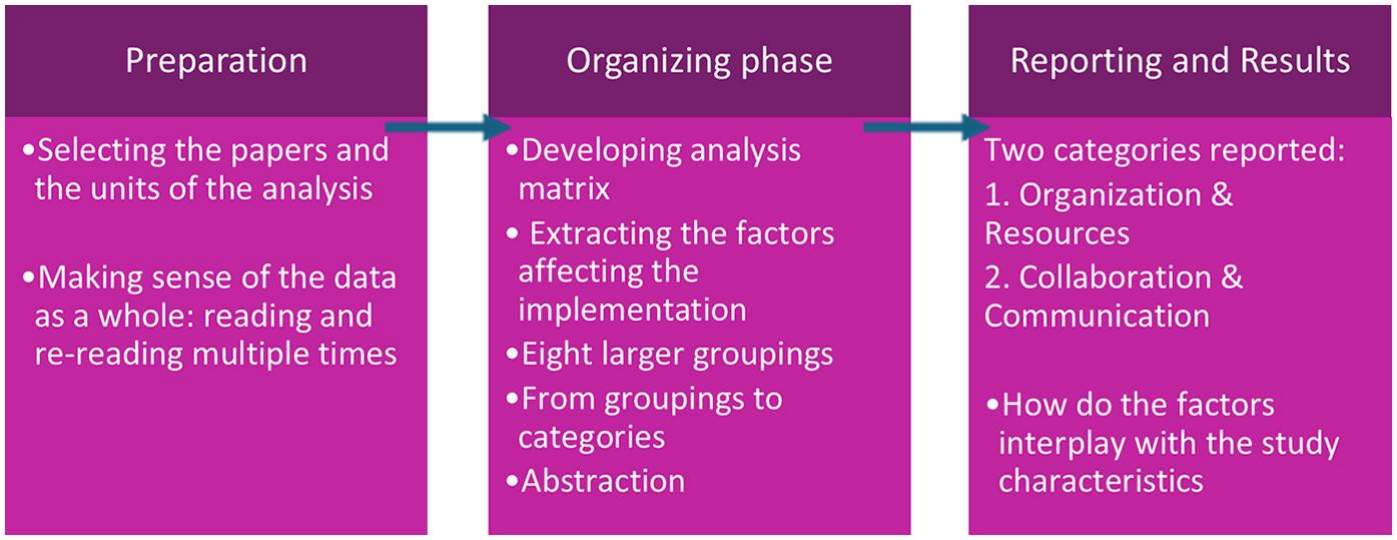
Phases in the content analysis.

The content analysis with a deductive-inductive approach inspired by Elo & Kyngäs (29) was applied. The analysis was divided into three phases: preparation, organizing, and reporting. See Figure 1. The content analysis aims to attain a condensed and broad description of factors influencing the implementation, with categories describing this (29). During the preparation phase, reading the papers, it became evident that factors influencing the implementations process were distributed across various sections in each paper. These factors were identified as either facilitators, barriers or factors with an unspecified impact. All relevant factors were extracted grouped into subcategories. These subcategories were then refined into main categories. To ensure validity, the categorization process involved iterative comparison between individual factors and the emerging categories. Factors that were not clearly labeled in terms of their influence on the implementation were excluded from the results, as their impact remains unclear. The main categories for the factors acting as facilitators or barriers are depicted in the “results” section.

## Results

The presentation of the results is as follows: a description of the study characteristics, including study selection, design, organizational approach, population, setting, and framework. This will be followed by a presentation of the factors influencing implementation in two categories: Organization & Resources and Collaboration & Communication.

### Study selection

**Figure 2:**
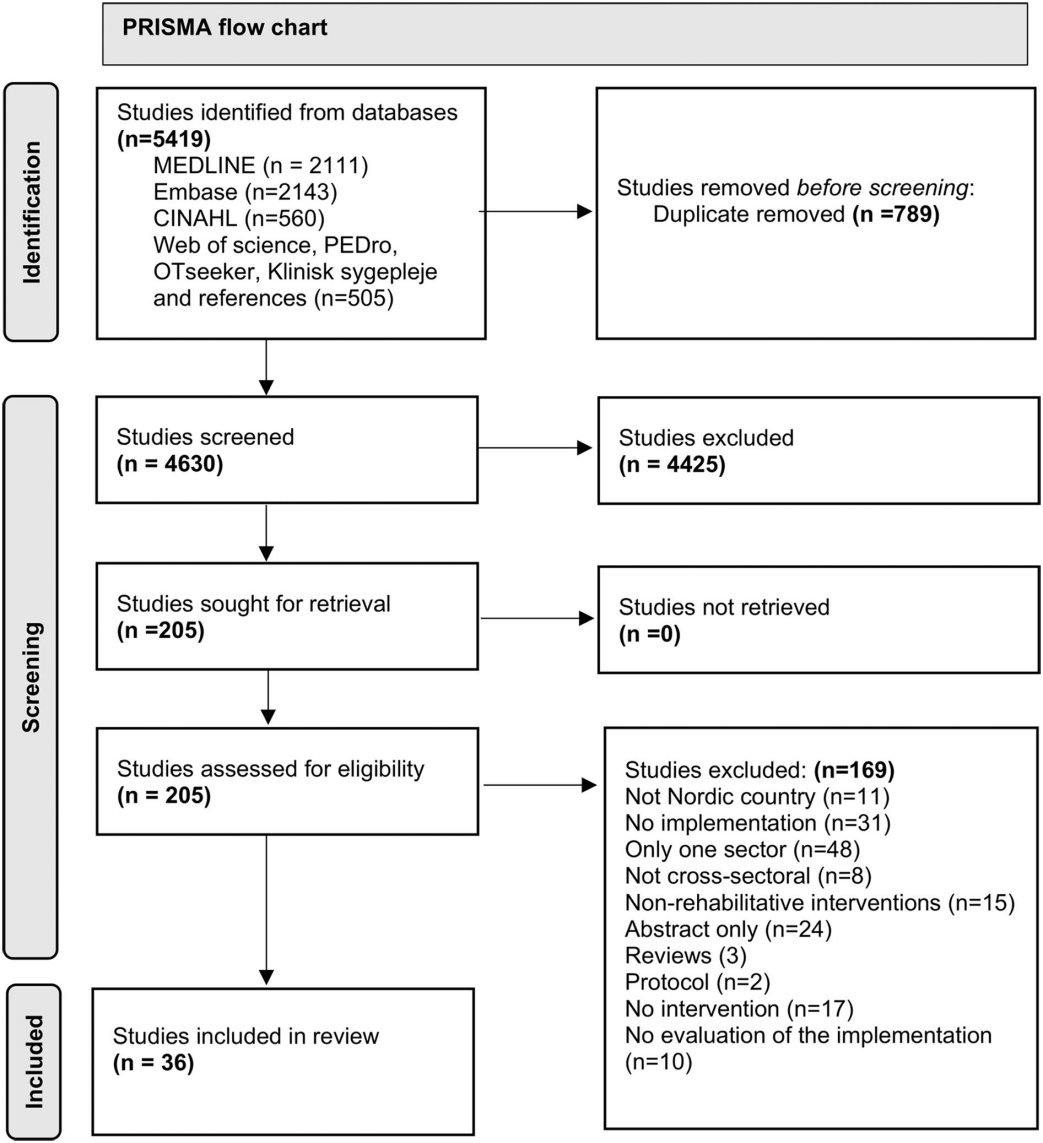
The PRISMA flow chart.

The search took place on November 1, 2023. Initially, 4,630 citations were screened, with 4,425 deemed ineligible. We conducted full-text screening on 205 papers, of which 169 were excluded. Ultimately, 36 papers were included in the final stage (27, 28, 30–63). The primary reason for exclusion at this stage was that the studies were either only abstracts, only focused on one sector, or the studies lacked a focus on implementation. See Figure 2 The PRISMA flow chart.

### Study characteristics and design

The 36 papers included were published from 2000 to 2023, with 28 published from 2014 and onwards. Interventions were allocated across four of the five Nordic countries. Denmark (*n* = 15) (32, 33, 36, 39, 41, 45, 48–50, 56–58, 61–63), Sweden (*n* = 13) (27, 31, 34, 35, 37, 38, 40, 42, 44, 46, 47, 51, 54), Norway (*n* = 7) (28, 43, 52, 53, 55, 59, 60), and Finland (*n* = 1) (30). No studies were identified from Iceland. In terms of study design, six were quantitative, twenty qualitative, and ten employed mixed methods. Some papers reported results from the same original intervention but with different aims (37, 38, 46, 47, 59, 60).

### Characteristics of the study participants

All interventions ultimately aimed to improve patient outcomes, but the interventions targeted various professions, which then influenced patient rehabilitation. In 28 studies, healthcare workers performed the implementation. The healthcare workers included were physiotherapists, occupational therapists, dieticians, psychologists, nurses, and nurses' assistants. A broad variation of other professions involved in implementation were physicians, general practitioners, pharmacists, managers/leaders, employment specialists, social insurance officers, discharge planning coordinators and researchers. Few studies included the patient as a part of the implementation process. Six studies included patient interviews as part of the evaluation (44, 48, 50, 59, 62), and two studies used questionnaires for patient evaluation (39, 41). A total of 29 studies did not ask for patients' opinions on how they were affected. See supplementary File S2.

### Description of sectors for the implementation

Twenty-two papers (28, 32, 35, 39, 41, 43–51, 55, 56, 58–63) described interventions moving from the secondary sector to the primary sector, while only one paper described an intervention moving from the primary sector to the secondary sector (27). The secondary sector refers to hospitals of varying sizes, often involving one or more wards. The primary sector encompasses a wide range of settings, including in- and outpatient rehabilitation units, home care, and specialized units.

Thirteen papers described an intervention with more parallel characteristics, implemented in either two sectors (30, 33, 37, 38, 40, 42, 52, 54, 57) or within two entities of a single sector (31, 34, 36, 53). The implementation was presented as more parallel than integrated across sectors, without any obvious element bridging them. Eight of the 13 papers described parallel interventions in the primary and secondary sectors (30, 33, 37, 38, 40, 52, 54, 57), e.g., a screening tool for anxiety and depression being implemented simultaneously in both a hospital and a municipality setting (33). Five of the 13 papers described interventions implemented only within the primary sector (31, 34, 36, 42, 53), but from one entity to another within the sector, for example in a municipality setting between a mental health care unit and a vocational service unit (42).

### Organizational approach to the implementation process

In 12 papers, the organizational approach to the implementation process was described as top-down (27, 30–32, 37, 39–42, 46, 52, 62). Three papers described a bottom-up approach (28, 44, 51), while two papers combined the two approaches (38, 54). Nineteen papers did not specify the approach (33–36, 43, 45, 47–50, 53, 55–61, 63). With regard to which organizational approach facilitates more successful implementation, all three papers employing a bottom-up approach report only limited success (28, 44, 51). As one paper highlights, “bottom-up process and enthusiasm are not enough” (p.8, Røsstad, 2015) (28). Conversely, top-down approaches appear to support the achievement of greater implementation success (27, 31, 32, 39, 41).

### Frameworks

To examine whether utilizing a framework facilitates implementation, we searched the papers for the use of frameworks. Seventeen papers utilize implementation frameworks considering both context and individual perspectives. See supplementary File S2—data extraction chart. The frameworks are RE-AIM (30, 56), PARiHS (35, 49), Normalization Process Theory (28, 33), ERIC (38), CFIR (37), Model of strategic change management developed by Pettigrew and Whipp (31), Realistic Evaluation (63), Framework by Nielsen et al. (47), implementation outcomes described by Proctor et al. (40, 52), The Theoretical Domains Framework (61), Process evaluation by Saunders et al. (34, 62) and Process evaluation by Thorne (36).

Only one of the papers described using an implementation framework for both planning and evaluating the intervention (56). The majority of the papers employ a framework for evaluation only (30, 33, 35, 36, 40, 63) or for data analysis (28, 37, 38, 47, 61, 62). The remaining papers utilize the implementation framework for a theoretical framework (31, 49) or to structure or inspire the interview guide (34, 52). A search through the 36 papers' results and conclusions sections did not convey more success using a framework.

### Factors influencing implementation

**Figure 3:**
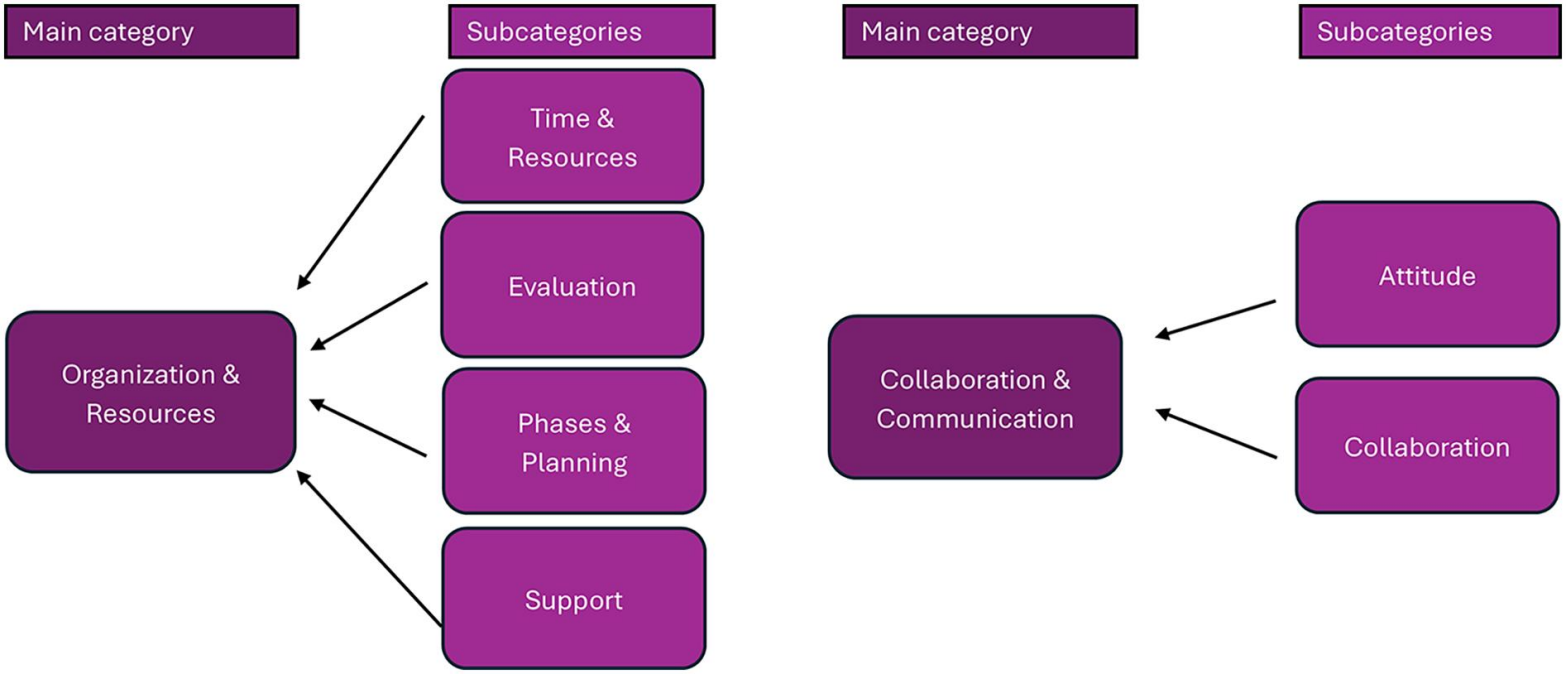
Categories and sub-categories of factors influencing the implementation.

Factors reported as facilitators, barriers, or both were grouped into subcategories during analysis and later organized into two main categories: *Organization & Resources* and *Collaboration & Communication* (see Figure 3). Only the main categories are described narratively, while subcategories are shown in Figure 3 to illustrate the analytical process.

The category of Organization & Resources encompasses factors that affect the implementation within the organizations, such as organizational structure, the teams and leadership roles, the operational procedures, and human resources, such as staffing and management. Financial resources, IT systems, and resource allocation are also in this category. As are the more evaluating factors.

Collaboration & Communication encompasses how teams, departments, and sectors work together, internal and external communication, and interaction with other stakeholders in the implementation. It also includes structures for discussions and decision-making, as well as other tools and strategies for a collaborative work environment.

### Organization & resources

*The alignment or especially misalignment* of the intervention within the context is a frequently mentioned hindering factor. It is attributed to, e.g., inflexible policies, the structure of the intervention, the size of the workplaces, a patient population that differs from what the intervention requires (27, 30, 31, 33–36, 39, 42, 44, 49, 52, 53, 63) or the organizations working together differing in aims and perspectives (43, 45, 50, 62). A facilitator that can affect this factor is pilot testing or trialability, mentioned by several papers as part of the pre-implementation phases (30, 37). Additionally, *involvement from the frontline personnel* at all stages is a crucial facilitating factor for the implementation and for the intervention to be integrated and contextually appropriate (32, 33, 38, 46, 49, 50, 53, 56, 57, 61).

*Time & Resources* are factors of great influence, both acting as barriers and facilitators to the implementation process. Commonly cited barriers arise in the form of a lack of resources, high staff turnovers, and time-consuming tasks like forming new teams or recruiting new staff (28, 31, 37, 40, 44, 51, 54, 56, 57, 62). Facilitators are commonly seen in the form of management engagement, where management is often responsible for providing additional resources, such as adjusting task lists, supporting staff in dedicating the required time, and allocation and helping to prioritize time (28, 37, 56, 61, 62).

*The time* factor can act as a facilitator when there is sufficient time for planning and conducting the implementation (48, 61, 63) and as a barrier when time is perceived as insufficient (27, 28, 30, 31, 35, 36, 39, 40, 46–49, 52). Insufficient time can lead to a feeling of inadequate preparation among implementers and uncertainty about what to expect (28, 34, 36, 46, 53, 55) and can impact the ability to reflect on challenges, causing feelings of professionalism to be compromised (35, 36).

*The workplace* itself is a factor. A barrier is a workplace culture resistant to change (34, 36, 39, 42, 49). Support or encouragement from the workplace, including project leads, leaders, and colleagues, can act as a barrier when absent (35, 36, 60) and as a facilitator when present (47, 63). Additional facilitating support encompasses technical, organizational, and emotional assistance (47, 48). Having a person designated as the facilitator on-site (49) can aid in creating the sense of being part of a larger common agenda (49). Researchers can facilitate implementation by providing the right research evidence that aligns with the clinical practice needs (35). Project leads or trainers contribute to a barrier through insufficient informal workplace training, insufficient continuous support, and a lack of follow-up (33, 63).

**Figure 4:**
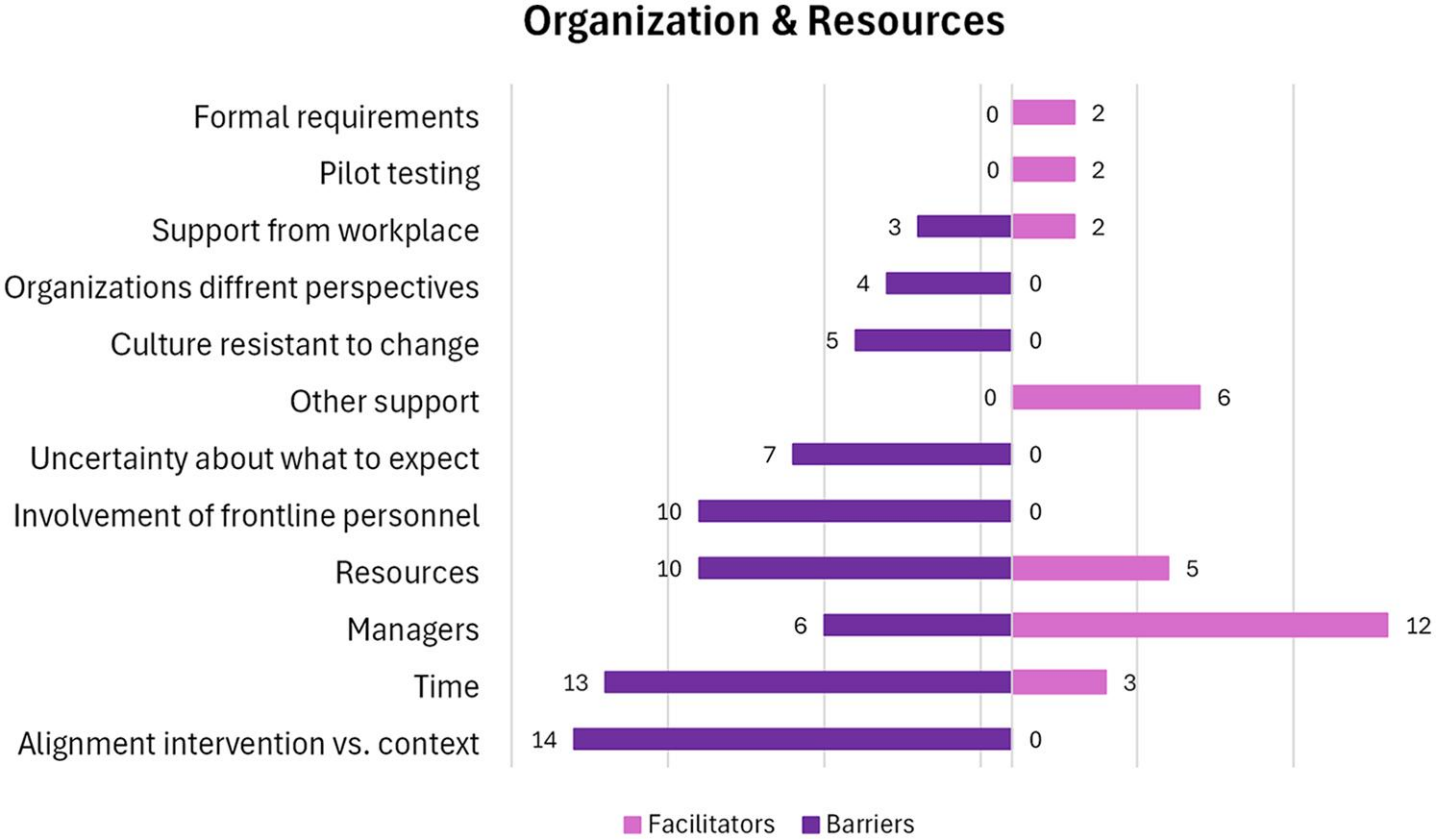
Number of papers within each factor in the category “Organization & Resources”. A visual presentation of the number of papers that holds facilitators and barriers within each factor.

*Managers* play a key role in the implementation process. They can be a barrier in the form of lack of support, due to absence from daily practice, or failing in providing leadership (33, 39, 46, 53, 62, 63). On the other hand, managers are frequently mentioned as facilitators (28, 33–35, 49, 53, 54, 56, 60–63). Facilitating management involves showing commitment and drive, being encouraging and responsive, as well as being realistic and concise. Formal requirements and mandatory participation facilitate implementation (33, 62). The factors and the number of papers that include each factor are depicted in Figure 4.

### Collaboration & communication

*Knowledge and competence* among staff regarding the intervention are identified as a factor that is a barrier to implementation when there is a lack of knowledge and a feeling of lack of competence towards the intervention (31–33, 37, 44, 49, 55) or lack of knowledge of each other's work (42, 56, 62). However, it can be a facilitator through educational activities (33–35, 38, 52, 54, 62, 63), including workshops, meetings, and education on relevant evidence. Knowledge sharing between staff at meetings or audits was a valuable resource; discussions and communication made the interventions meaningful for staff (31, 34, 35, 39, 49, 53, 56, 57, 59, 61), as well as training in a real environment (35, 47, 52, 63), and learning through their own experience (33, 35, 55, 63). These activities helped staff feel prepared to implement the intervention.

*Attitudes among staff* are a factor that holds both barriers and facilitators. Attitudes can be a barrier when there is a lack of motivation, a perception of no need for change or resistance towards change, and skepticism towards other professions and their capabilities that complicate collaboration (31, 34, 39, 42, 44, 45, 50, 54). Attitude is a facilitating factor when it includes mutual interest, active engagement from all parties, increasing awareness, positive expectations, respect towards the practitioners’ roles from managers and researchers, and a sense of responsibility towards practicing improvement (31, 33–36, 41, 46, 54, 56, 57, 61, 62).

*Communication* is a factor that can facilitate collaboration (36, 39, 45–47, 54, 60, 62). This includes more structured communication, guidelines, and communication at all levels within and across organizations. The setting for communication must be developed in the specific context of cross-sectoral work. This is also the case for documentation flow and systematic processes, such as referrals that were a facilitator when functioning (34, 35, 39, 41, 43, 44, 52, 53, 62) and an important factor for cross-sectoral communication. Working towards a shared goal can be both facilitating when it occurs (34, 39) and a barrier when not (44, 45, 50, 54). Communication can be a barrier when respect is lacking, e.g., researchers have to respect stakeholders' knowledge and expertise in their respective fields (35, 59).

**Figure 5:**
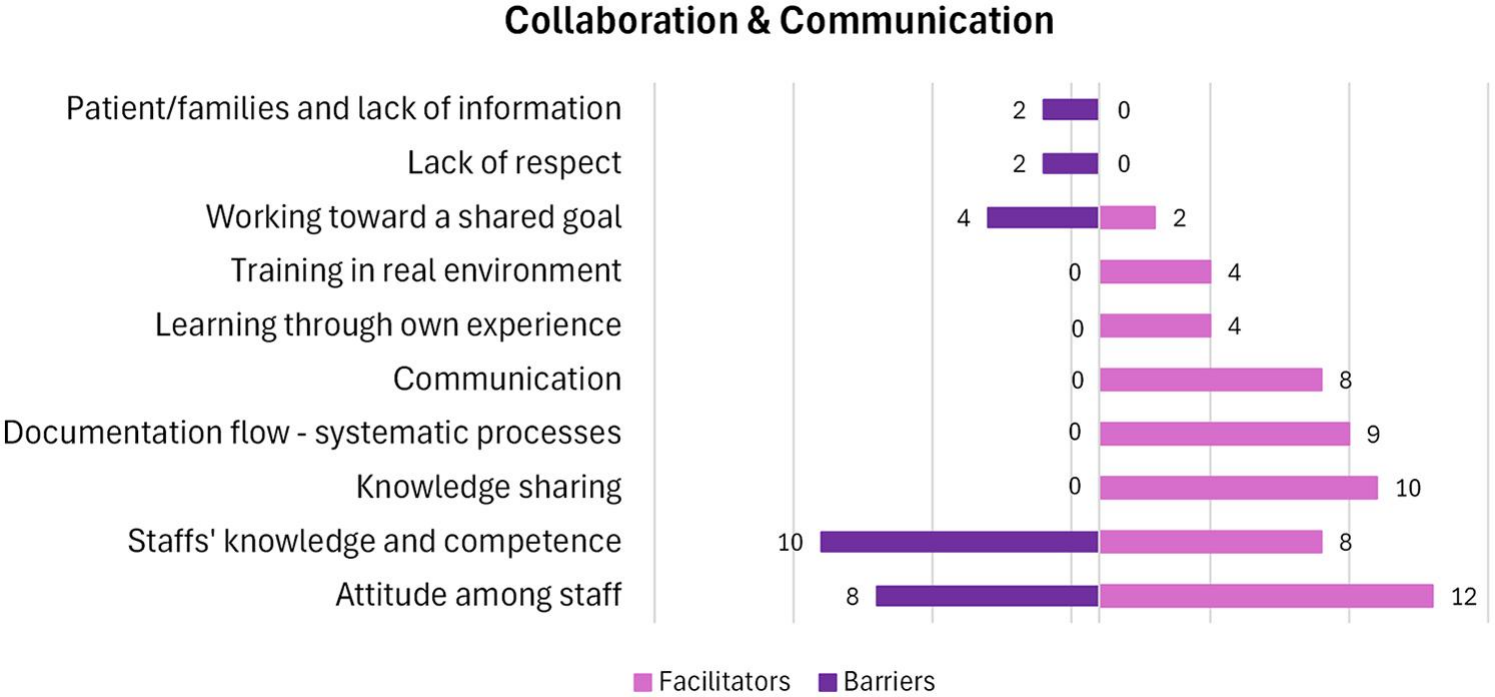
Number of papers within each factor in the category “Collaboration & Communication”. A visual presentation of the number of papers that hold facilitators and barriers within each factor.

*Patients and their families* can be a factor that serves as a barrier when they are dissatisfied with the information provided, as it was either repetitive or contradictory to previous information received (33, 63). The factors and the number of papers that include each factor are depicted in Figure 5.

## Discussion

This scoping review examined existing research on the cross-sectoral implementation of rehabilitation in Nordic health systems, focusing on barriers and facilitators that influence the implementation processes. Synthesizing the research reveals that most interventions reported took place during patients' transitions from hospital to primary care in Denmark, Sweden, and Norway. Results consist of a) the characteristics of the studies as to the country, implementers, sectors, organizational approach and frameworks and b) categories of factors that can affect the implementation process: Organization & Resources and Collaboration & Communication.

Considering the PICO elements guiding the review, the included studies addressed broad *Populations*, typically adults with varying rehabilitation needs, though details on diagnosis and impairments were inconsistently reported. The *Interventions* ranged from exercise and person-centered care to discharge planning, screening tools, and structured rehabilitation programs. Most were implemented in cross-sectoral *Contexts/Settings*, particularly transitions from secondary (hospital) to primary or community care, while others described more parallel sectoral initiatives. The *Outcomes* most often reported related to implementation processes—such as feasibility, staff engagement, and organizational alignment, whereas direct patient outcomes and perspectives were less frequently included. This imbalance suggests that while organizational and professional experiences are well-documented, patients' needs, goals, and barriers to service use remain underexplored (64).

Several findings warrant further discussion. Notably, most of the studies included were published from 2014 onwards. This trend aligns with a corresponding increase in the scholarly use of the term *implementation*, as evidenced by a PubMed search (65) showing a doubling of such articles between 2014 and 2024. This trend may be interpreted in light of the growing focus in implementation research in general, an emphasis on rehabilitation service's needs, and the recognition of persistent challenges in translating evidence into clinical practice (66). Bunger et al. (67) similarly underscore the necessity for more precise and systematic reporting of implementation strategies to facilitate replicability and cumulative knowledge development within the field. Surprisingly, our review did not identify a common or standardized framework for conducting or reporting implementation processes across the included papers. The absence of such structured approaches further highlights the pressing need for the establishment of standardized methodologies to enhance comparability, rigor, and transparency in future research endeavors.

Our findings show a top-down organizational approach to implementation is most frequently applied and appears to be more facilitating than a bottom-up approach. Top-down processes seem to succeed particularly when they involve mandatory participation or strong leadership characterized by visible support, commitment, and realistic goal setting, while also ensuring the involvement of front-line personnel and stakeholders. This reliance on top-down strategies has important implications for policy design. It suggests that centralized leadership and formal structures may provide the necessary authority and resources to drive cross-sectoral implementation but also raises concerns about flexibility and ownership at the local level. Bottom-up approaches, although less frequently reported as successful, may still be viable under conditions where front-line staff are well-prepared, supported, and empowered to adapt interventions to local contexts. Co-designing strategies, as highlighted by Manalili et al. (68), or a more inclusive approach that engage front-line personnel early (65, 66), may help bridge the gap between top-down directives and bottom-up ownership. Without adequate preparation however, involvement of front-line personnel can hinder rather than support implementation, as shown when healthcare professionals are unprepared for new roles and transitions (67). The focus on context during implementation is highlighted by Skivington et al. in the Medical Research Council (MRC) framework of complex interventions (14), by van Scherpenseel et al. (69), and in The Implementation in Context (ICON) framework by Squires et al., which has a specific focus on the importance of analyzing the context of the intervention (17). Unexpectedly, when a comparison of the results of this scoping review was made with the core elements in the MRC (14) and ICON frameworks (17), we found that there was a lack of focus on adapting or refining the implementation throughout the implementation period. Although the results in this scoping review do highlight some importance of interorganizational relationships, these relationships should play an even greater role in cross-sectoral implementation according to the ICON framework (18).

Another major gap identified in the comparison was the limited involvement of stakeholders, particularly patients. Consequently, most studies included in this scoping review concentrate on the intervention and evaluation of healthcare personnel, with only a few incorporating the patients' perspectives. Patient involvement is widely recognized as essential across all research settings (70, 71). Therefore, it is a significant limitation that these studies do not report on patient involvement, and the potential impact on the implementations remains unexplored. The lack of patient involvement contradicts the more inclusive practice by the MRC, the ICON framework and Eskerod et al.'s (72) project management theory. The last which holds the concept of 'stakeholder inclusiveness', which entails recognizing and addressing the needs and concerns of all stakeholders, regardless of their level of influence or potential impact on the project (72).

Aligning with Proctor et al. (73), who argue that the strategic application of frameworks or other systematic approaches can enhance implementation success, we searched the papers for the use of implementation frameworks. Notably, the seventeen interventions which employed an implementation framework that accounted for both contextual factors and individual perspectives, primarily used it for evaluation purposes rather than for planning or guiding the implementation process. This limited use may be attributed to the wide variety of available frameworks and the lack of consensus on which to apply (17, 74). An emphasis on the early phases of implementation is advocated by Alley et al. (75), as to a thorough investigation of stakeholder engagement, feasibility, and organizational readiness during the pre-implementation phase, factors that according to Alley et al. (75) will significantly enhance the likelihood of successful implementation. There should be a focus on strengthening this in future implementation processes.

### Strengths and limitations

We consider the broad scope of rehabilitation focused on care, physical, mental, and/or social outcomes a strength in our effort to identify factors affecting cross-sectoral implementation. The papers in this scoping review, although covering various rehabilitative areas, all contribute to valuable knowledge for future implementations efforts. A strength of this study is that we not only searched in the larger databases but also in databases targeted at rehabilitation, such as PEDro, OTseeker, and journal of Klinisk Sygepleje (Clinical Nursing), and in the search for relevant papers, included a citation search in Web of Science. Also, we adhere to the PRISMA-ScR recommendations and reporting standards, the consultation with information specialists in deciding the search strategy, and the use of multiple reviewers in all phases.

This review has limitations. Firstly, despite using an extensive search strategy, we may have missed some publications as the research papers do not always clearly describe the setting or focus on implementation and a more precise and established terminology could have increased the number of relevant studies found. However, the potential loss of relevant studies was minimized by hand-screening for additional records as well as by using Web Of Science. Secondly, only published studies were included, and grey literature and unpublished studies were excluded. While this decision supported feasibility and transparency of the review process, it may have biased the findings toward academic and clinical perspectives, underrepresenting practice-based or policy-level insights. Thirdly, sector-specific studies were excluded, which means that contextual factors unique to single-sector settings were not captured. Whether these differ substantially from those influencing cross-sectoral implementation is unknown and warrants further research. Fourthly, a third of the included studies, despite having a cross-sectoral design, described a more parallel implementation without a clear element bridging sectors. These studies are presented and included. Thus, the facilitators and barriers for cross-sectorial implementation are still experienced in a multiple-sector setting. Several factors that seem to affect the implementation were not labelled or distinguished by the authors as to what effect they had on the implementation and could possibly hold valuable knowledge. Finally, consistent with scoping review methodology, we did not appraise the quality of the studies included. This lack of critical appraisal means the strength of evidence underlying individual findings cannot be assessed within this review. Readers interested in evaluating the robustness of particular studies are therefore encouraged to explore the articles included independently.

## Conclusion

Evidence from this scoping review shows that the two most reported factors influencing implementation are lack of time and resources (Organization & Resources) and staff attitudes and competencies (Collaboration & Communication). Other barriers include poor alignment between interventions and context, limited frontline involvement, negative staff attitudes, and lack of knowledge. Facilitators include strong managerial support, engaged stakeholders, knowledge sharing, and effective communication and documentation flows. The knowledge presented in this review is drawn from studies conducted in Denmark, Sweden, and Norway, with no eligible studies identified from Finland or Iceland.

Based on these findings, four key recommendations for implementing cross-sectoral rehabilitation can be made:

1. Allocate resources and time to adequately prepare for implementation and support sustainability.
2. Ensure strong and committed leadership, ideally with clear top-down support, to drive and coordinate the process.
3. Involve stakeholders early, including frontline personnel, to build engagement, competence, and shared ownership.
4. Foster adaptive and context-sensitive processes, including systematic communication, shared documentation, and opportunities for dialogue and refinement.
