# Supplementary material for: Implementation of cross-sectoral rehabilitation in the Nordic countries: a scoping review
Source: Front Health Serv. 2025 Sep 19;5:1662230. doi: 10.3389/frhs.2025.1662230 (PMC12491184; doi:10.3389/frhs.2025.1662230)
Supplement: Supplementary file 1 [file Table1.docx]

**Supplementary file S1 – search strings**

[Ovid Medline search string 1](#_Toc1928074903)

[Ovid Embase search string 8](#_Toc1849580487)

[CINAHL (EBSCO host) search string 13](#_Toc1453279378)

[PEDro, OTseeker, Klinisk sygepleje (via Idun) 17](#_Toc1006962536)

# Ovid Medline search string

November 1, 2023

| **#** | **Query** |
| --- | --- |
| 1 | "scandinavian and nordic countries"/ or denmark/ or greenland/ or norway/ or svalbard/ or sweden/ or finland/ or iceland/ |
| 2 | (denmark or danmark or sweden or sverige or norway or norge or finland or iceland).ab,in,kf,ti. |
| 3 | (denmark* or dane* or danish or sweden* or swede* or swedish or norway* or norwegian* or finland or finn* or iceland or icelandic* or greenland or greenlandic* or scandinavia* or nordic*).ab,kf,ti. |
| 4 | or/1-3 |
| 5 | exp Exercise/ |
| 6 | exp Health Education/ |
| 7 | exp Physical Therapy Modalities/ |
| 8 | exp Rehabilitation/ |
| 9 | (physical* adj3 activit*).ab,kf,ti. |
| 10 | exp motor activity/ or social behavior/ or exp motivation/ |
| 11 | exp Sedentary Behavior/ |
| 12 | exp Self-Control/ |
| 13 | activit* of daily living.ab,kf,ti. |
| 14 | exp exercise therapy/ or occupational therapy/ or recreation therapy/ |
| 15 | "Recovery of Function"/ |
| 16 | Leisure Activities/ |
| 17 | health behavior/ or health risk behaviors/ |
| 18 | (Physiotherapies or physiotherapy).ab,kf,ti. |
| 19 | (Occupational therapy or occupational therapies).ab,kf,ti. |
| 20 | (rehab* adj5 care).ab,kf,ti. |
| 21 | "exercise*".ab,kf,ti. |
| 22 | exergaming.ab,kf,ti. |
| 23 | "gymnastic*".ab,kf,ti. |
| 24 | "run*".ab,kf,ti. |
| 25 | "swim*".ab,kf,ti. |
| 26 | "walk*".ab,kf,ti. |
| 27 | "jog*".ab,kf,ti. |
| 28 | sprint*.ab,kf,ti. |
| 29 | (physical workout or physical work-out).ab,kf,ti. |
| 30 | "stair climbing".ab,kf,ti. |
| 31 | "health education".ab,kf,ti. |
| 32 | "health fair* ".ab,kf,ti. |
| 33 | "health promotion* ".ab,kf,ti. |
| 34 | (education and (breast feeding or childbirth or death or dental health or diabetes or hiv or nutrition or parenting or patient or preoperative or psycho or school health or self care or sleep)).ab,kf,ti. |
| 35 | "sex education".ab,kf,ti. |
| 36 | "smoking prevention* ".ab,kf,ti. |
| 37 | "health literacy".ab,kf,ti. |
| 38 | "healthy people program* ".ab,kf,ti. |
| 39 | "prenatal education".ab,kf,ti. |
| 40 | "physical therap* modalit* ".ab,kf,ti. |
| 41 | "animal assisted therap* ".ab,kf,ti. |
| 42 | "equine assisted therap* ".ab,kf,ti. |
| 43 | "Dry needling".ab,kf,ti. |
| 44 | "electric stimulation therap* ".ab,kf,ti. |
| 45 | electroacupuncture.mp. or electro acupuncture.ab,kf,ti. |
| 46 | "pulsed radiofrequency treatment".ab,kf,ti. |
| 47 | "spinal cord stimulation".ab,kf,ti. |
| 48 | Qigong.ab,kf,ti. |
| 49 | ("tai ji" or "tai chi").ab,kf,ti. |
| 50 | yoga.ab,kf,ti. |
| 51 | ("blood flow restriction therap*" or "blood flow restriction training").ab,kf,ti. |
| 52 | ("motion therap* " or "movement therap*").ab,kf,ti. |
| 53 | "extracorporeal shockwave therap* ".ab,kf,ti. |
| 54 | "hydrotherap*".ab,kf,ti. |
| 55 | "aquatic therap* ".ab,kf,ti. |
| 56 | "motion therap* ".ab,kf,ti. |
| 57 | "therap*, soft tissue ".ab,kf,ti. |
| 58 | massage.ab,kf,ti. |
| 59 | "manual lymphatic drainage".ab,kf,ti. |
| 60 | "art therap* ".ab,kf,ti. |
| 61 | "bibliotherap*".ab,kf,ti. |
| 62 | "early ambulation* ".ab,kf,ti. |
| 63 | "motor activit* ".ab,kf,ti. |
| 64 | ("social behavior" or "social behaviour").ab,kf,ti. |
| 65 | ("cooperative behavior" or "cooperative behaviour").ab,kf,ti. |
| 66 | empowerment.ab,kf,ti. |
| 67 | motivation.ab,kf,ti. |
| 68 | "achievement*".ab,kf,ti. |
| 69 | "aspirational".ab,kf,ti. |
| 70 | ("sedentary behavior" or "sedentary behaviour").ab,kf,ti. |
| 71 | "self-control".ab,kf,ti. |
| 72 | "emotion* regulation".ab,kf,ti. |
| 73 | "recovery of function* ".ab,kf,ti. |
| 74 | "leisure activit* ".ab,kf,ti. |
| 75 | (sport* adj3 rehab*).ab,kf,ti. |
| 76 | recreation*.ab,kf,ti. |
| 77 | dancing.ab,kf,ti. |
| 78 | "hobb*".ab,kf,ti. |
| 79 | horticulture.ab,kf,ti. |
| 80 | gardening.ab,kf,ti. |
| 81 | hunting.ab,kf,ti. |
| 82 | "game*".ab,kf,ti. |
| 83 | "athletic performance".ab,kf,ti. |
| 84 | fitness.ab,kf,ti. |
| 85 | "physical endurance".ab,kf,ti. |
| 86 | (baseball or basketball or bicycl* or boxing or cricket or football or golf or hockey or "martial arts" or mountaineering or tennis or rugby or skating or skiing or soccer or "track and field" or volleyball or diving or "weight lifting" or wrestling).ab,kf,ti. |
| 87 | ("health behavior" or "health behaviour").ab,kf,ti. |
| 88 | ("health risk behavior*" or "health risk behavour*").ab,kf,ti. |
| 89 | kinesiotherap*.ab,kf,ti. |
| 90 | physical effort*.ab,kf,ti. |
| 91 | physical exertion.ab,kf,ti. |
| 92 | calisthenics.ab,kf,ti. |
| 93 | cool down.ab,kf,ti. |
| 94 | pilates.ab,kf,ti. |
| 95 | "plyometric*".ab,kf,ti. |
| 96 | squatting.ab,kf,ti. |
| 97 | warm up.ab,kf,ti. |
| 98 | physical therapy.ab,kf,ti. |
| 99 | chest wall oscillation*.ab,kf,ti. |
| 100 | joint mobilization*.mp. or joint mobilisation*.ab,kf,ti. |
| 101 | kinesio taping.ab,kf,ti. |
| 102 | (resocialisation or resocialization or revalidation).ab,kf,ti. |
| 103 | bladder training.ab,kf,ti. |
| 104 | community reintegration.ab,kf,ti. |
| 105 | constraint induced therap*.ab,kf,ti. |
| 106 | drug dependence treatment*.ab,kf,ti. |
| 107 | functional assessment*.ab,kf,ti. |
| 108 | mental health recovery.ab,kf,ti. |
| 109 | "mirror therap*".ab,kf,ti. |
| 110 | "sociotherap*".ab,kf,ti. |
| 111 | (recreational therapy or recreational therapies).ab,kf,ti. |
| 112 | sensorimotor integration.ab,kf,ti. |
| 113 | (patient* adj5 "self report").ab,kf,ti. |
| 114 | (incentive adj5 behavio*).ab,kf,ti. |
| 115 | "kinesitherap*".ab,kf,ti. |
| 116 | "kinesiotherap*".ab,kf,ti. |
| 117 | sedentary lifestyle.ab,kf,ti. |
| 118 | Convalescence/ |
| 119 | convalescence.ab,kf,ti. |
| 120 | "rehabilit*".ab,kf,ti. |
| 121 | "training*".ab,kf,ti. |
| 122 | or/5-121 |
| 123 | exp "Continuity of Patient Care"/ |
| 124 | (integrat* adj3 care).ab,kf,ti. |
| 125 | "patient* hand off".ab,kf,ti. |
| 126 | "patient* handoff".ab,kf,ti. |
| 127 | "patient* transition*".ab,kf,ti. |
| 128 | inter sector*.mp. or intersector*.ab,kf,ti. |
| 129 | (multi sector* or multisector* or multi-sector*).ab,kf,ti. |
| 130 | (continue* adj3 care).ab,kf,ti. |
| 131 | clinical handover.ab,kf,ti. |
| 132 | (patient* adj3 transfer*).ab,kf,ti. |
| 133 | (patient* adj3 pathway*).ab,kf,ti. |
| 134 | (patient* adj3 trajector*).ab,kf,ti. |
| 135 | (cross sector* or cross-sector* or across sector* or a cross sector* or between sector*).ab,kf,ti. |
| 136 | (discharg* adj3 (home or hospital* or center* or centre or clinic*)).ab,kf,ti. |
| 137 | "Delivery of Health Care, Integrated"/ |
| 138 | delivery of health care.ab,kf,ti. |
| 139 | or/123-138 |
| 140 | exp Health Planning/ |
| 141 | "health planning".ti,ab,kf. |
| 142 | "Health Care Reform".ti,ab,kf. |
| 143 | "Health Planning Guidelines".ti,ab,kf. |
| 144 | "Health Priorities".ti,ab,kf. |
| 145 | "Health Resources".ti,ab,kf. |
| 146 | "Resource-Limited Settings".ti,ab,kf. |
| 147 | "Health Impact Assessment*".ti,ab,kf. |
| 148 | Implementation Science/ or Translational Research, Biomedical/ |
| 149 | exp Social Planning/ |
| 150 | "social planning".ti,ab,kf. |
| 151 | Quality Improvement/ or Program Development/ |
| 152 | "quality of health care"/ or "outcome and process assessment, health care"/ or quality assurance, health care/ |
| 153 | (Implementation Science or Translational Research).ab,kf,ti. |
| 154 | "quality improvement*".ab,kf,ti. |
| 155 | quality of health care.ab,kf,ti. |
| 156 | (outcome assessment or process assessment).ab,kf,ti. |
| 157 | quality assurance.ab,kf,ti. |
| 158 | (evaluat* adj3 (programme or program or programs)).ti,ab,kf. |
| 159 | Implement*.ti,ab,kf. |
| 160 | (evaluat* adj3 project*).ti,ab,kf. |
| 161 | program development.ab,kf,ti. |
| 162 | or/140-161 |
| 163 | 4 and 122 and 139 and 162 |
| 164 | "scandinavian and nordic countries"/ or denmark/ or greenland/ or norway/ or svalbard/ or sweden/ or finland/ or iceland/ |
| 165 | (denmark or danmark or sweden or sverige or norway or norge or finland or iceland).ab,in,kf,ti. |
| 166 | (denmark* or dane* or danish or sweden* or swede* or swedish or norway* or norwegian* or finland or finn* or iceland or icelandic* or greenland or greenlandic* or scandinavia* or nordic*).ab,kf,ti. |
| 167 | or/164-166 |
| 168 | exp Exercise/ |
| 169 | exp Health Education/ |
| 170 | exp Physical Therapy Modalities/ |
| 171 | exp Rehabilitation/ |
| 172 | (physical* adj3 activit*).ab,kf,ti. |
| 173 | exp motor activity/ or social behavior/ or exp motivation/ |
| 174 | exp Sedentary Behavior/ |
| 175 | exp Self-Control/ |
| 176 | activit* of daily living.ab,kf,ti. |
| 177 | exp exercise therapy/ or occupational therapy/ or recreation therapy/ |
| 178 | "Recovery of Function"/ |
| 179 | Leisure Activities/ |
| 180 | health behavior/ or health risk behaviors/ |
| 181 | (Physiotherapies or physiotherapy).ab,kf,ti. |
| 182 | (Occupational therapy or occupational therapies).ab,kf,ti. |
| 183 | (rehab* adj5 care).ab,kf,ti. |
| 184 | "exercise*".ab,kf,ti. |
| 185 | exergaming.ab,kf,ti. |
| 186 | "gymnastic*".ab,kf,ti. |
| 187 | "run*".ab,kf,ti. |
| 188 | "swim*".ab,kf,ti. |
| 189 | "walk*".ab,kf,ti. |
| 190 | "jog*".ab,kf,ti. |
| 191 | sprint*.ab,kf,ti. |
| 192 | (physical workout or physical work-out).ab,kf,ti. |
| 193 | "stair climbing".ab,kf,ti. |
| 194 | "health education".ab,kf,ti. |
| 195 | "health fair* ".ab,kf,ti. |
| 196 | "health promotion* ".ab,kf,ti. |
| 197 | (education and (breast feeding or childbirth or death or dental health or diabetes or hiv or nutrition or parenting or patient or preoperative or psycho or school health or self care or sleep)).ab,kf,ti. |
| 198 | "sex education".ab,kf,ti. |
| 199 | "smoking prevention* ".ab,kf,ti. |
| 200 | "health literacy".ab,kf,ti. |
| 201 | "healthy people program* ".ab,kf,ti. |
| 202 | "prenatal education".ab,kf,ti. |
| 203 | "physical therap* modalit* ".ab,kf,ti. |
| 204 | "animal assisted therap* ".ab,kf,ti. |
| 205 | "equine assisted therap* ".ab,kf,ti. |
| 206 | "Dry needling".ab,kf,ti. |
| 207 | "electric stimulation therap* ".ab,kf,ti. |
| 208 | electroacupuncture.mp. or electro acupuncture.ab,kf,ti. |
| 209 | "pulsed radiofrequency treatment".ab,kf,ti. |
| 210 | "spinal cord stimulation".ab,kf,ti. |
| 211 | Qigong.ab,kf,ti. |
| 212 | ("tai ji" or "tai chi").ab,kf,ti. |
| 213 | yoga.ab,kf,ti. |
| 214 | ("blood flow restriction therap*" or "blood flow restriction training").ab,kf,ti. |
| 215 | ("motion therap* " or "movement therap*").ab,kf,ti. |
| 216 | "extracorporeal shockwave therap* ".ab,kf,ti. |
| 217 | "hydrotherap*".ab,kf,ti. |
| 218 | "aquatic therap* ".ab,kf,ti. |
| 219 | "motion therap* ".ab,kf,ti. |
| 220 | "therap*, soft tissue ".ab,kf,ti. |
| 221 | massage.ab,kf,ti. |
| 222 | "manual lymphatic drainage".ab,kf,ti. |
| 223 | "art therap* ".ab,kf,ti. |
| 224 | "bibliotherap*".ab,kf,ti. |
| 225 | "early ambulation* ".ab,kf,ti. |
| 226 | "motor activit* ".ab,kf,ti. |
| 227 | ("social behavior" or "social behaviour").ab,kf,ti. |
| 228 | ("cooperative behavior" or "cooperative behaviour").ab,kf,ti. |
| 229 | empowerment.ab,kf,ti. |
| 230 | motivation.ab,kf,ti. |
| 231 | "achievement*".ab,kf,ti. |
| 232 | "aspirational".ab,kf,ti. |
| 233 | ("sedentary behavior" or "sedentary behaviour").ab,kf,ti. |
| 234 | "self-control".ab,kf,ti. |
| 235 | "emotion* regulation".ab,kf,ti. |
| 236 | "recovery of function* ".ab,kf,ti. |
| 237 | "leisure activit* ".ab,kf,ti. |
| 238 | (sport* adj3 rehab*).ab,kf,ti. |
| 239 | recreation*.ab,kf,ti. |
| 240 | dancing.ab,kf,ti. |
| 241 | "hobb*".ab,kf,ti. |
| 242 | horticulture.ab,kf,ti. |
| 243 | gardening.ab,kf,ti. |
| 244 | hunting.ab,kf,ti. |
| 245 | "game*".ab,kf,ti. |
| 246 | "athletic performance".ab,kf,ti. |
| 247 | fitness.ab,kf,ti. |
| 248 | "physical endurance".ab,kf,ti. |
| 249 | (baseball or basketball or bicycl* or boxing or cricket or football or golf or hockey or "martial arts" or mountaineering or tennis or rugby or skating or skiing or soccer or "track and field" or volleyball or diving or "weight lifting" or wrestling).ab,kf,ti. |
| 250 | ("health behavior" or "health behaviour").ab,kf,ti. |
| 251 | ("health risk behavior*" or "health risk behavour*").ab,kf,ti. |
| 252 | kinesiotherap*.ab,kf,ti. |
| 253 | physical effort*.ab,kf,ti. |
| 254 | physical exertion.ab,kf,ti. |
| 255 | calisthenics.ab,kf,ti. |
| 256 | cool down.ab,kf,ti. |
| 257 | pilates.ab,kf,ti. |
| 258 | "plyometric*".ab,kf,ti. |
| 259 | squatting.ab,kf,ti. |
| 260 | warm up.ab,kf,ti. |
| 261 | physical therapy.ab,kf,ti. |
| 262 | chest wall oscillation*.ab,kf,ti. |
| 263 | joint mobilization*.mp. or joint mobilisation*.ab,kf,ti. |
| 264 | kinesio taping.ab,kf,ti. |
| 265 | (resocialisation or resocialization or revalidation).ab,kf,ti. |
| 266 | bladder training.ab,kf,ti. |
| 267 | community reintegration.ab,kf,ti. |
| 268 | constraint induced therap*.ab,kf,ti. |
| 269 | drug dependence treatment*.ab,kf,ti. |
| 270 | functional assessment*.ab,kf,ti. |
| 271 | mental health recovery.ab,kf,ti. |
| 272 | "mirror therap*".ab,kf,ti. |
| 273 | "sociotherap*".ab,kf,ti. |
| 274 | (recreational therapy or recreational therapies).ab,kf,ti. |
| 275 | sensorimotor integration.ab,kf,ti. |
| 276 | (patient* adj5 "self report").ab,kf,ti. |
| 277 | (incentive adj5 behavio*).ab,kf,ti. |
| 278 | "kinesitherap*".ab,kf,ti. |
| 279 | "kinesiotherap*".ab,kf,ti. |
| 280 | sedentary lifestyle.ab,kf,ti. |
| 281 | Convalescence/ |
| 282 | convalescence.ab,kf,ti. |
| 283 | "rehabilit*".ab,kf,ti. |
| 284 | "training*".ab,kf,ti. |
| 285 | or/168-284 |
| 286 | exp "Continuity of Patient Care"/ |
| 287 | (integrat* adj3 care).ab,kf,ti. |
| 288 | "patient* hand off".ab,kf,ti. |
| 289 | "patient* handoff".ab,kf,ti. |
| 290 | "patient* transition*".ab,kf,ti. |
| 291 | inter sector*.mp. or intersector*.ab,kf,ti. |
| 292 | (multi sector* or multisector* or multi-sector*).ab,kf,ti. |
| 293 | (continue* adj3 care).ab,kf,ti. |
| 294 | clinical handover.ab,kf,ti. |
| 295 | (patient* adj3 transfer*).ab,kf,ti. |
| 296 | (patient* adj3 pathway*).ab,kf,ti. |
| 297 | (patient* adj3 trajector*).ab,kf,ti. |
| 298 | (cross sector* or cross-sector* or across sector* or a cross sector* or between sector*).ab,kf,ti. |
| 299 | (discharg* adj3 (home or hospital* or center* or centre or clinic*)).ab,kf,ti. |
| 300 | "Delivery of Health Care, Integrated"/ |
| 301 | delivery of health care.ab,kf,ti. |
| 302 | or/286-301 |
| 303 | exp Health Planning/ |
| 304 | "health planning".ti,ab,kf. |
| 305 | "Health Care Reform".ti,ab,kf. |
| 306 | "Health Planning Guidelines".ti,ab,kf. |
| 307 | "Health Priorities".ti,ab,kf. |
| 308 | "Health Resources".ti,ab,kf. |
| 309 | "Resource-Limited Settings".ti,ab,kf. |
| 310 | "Health Impact Assessment*".ti,ab,kf. |
| 311 | Implementation Science/ or Translational Research, Biomedical/ |
| 312 | exp Social Planning/ |
| 313 | "social planning".ti,ab,kf. |
| 314 | Quality Improvement/ or Program Development/ |
| 315 | "quality of health care"/ or "outcome and process assessment, health care"/ or quality assurance, health care/ |
| 316 | (Implementation Science or Translational Research).ab,kf,ti. |
| 317 | "quality improvement*".ab,kf,ti. |
| 318 | quality of health care.ab,kf,ti. |
| 319 | (outcome assessment or process assessment).ab,kf,ti. |
| 320 | quality assurance.ab,kf,ti. |
| 321 | (evaluat* adj3 (programme or program or programs)).ti,ab,kf. |
| 322 | Implement*.ti,ab,kf. |
| 323 | (evaluat* adj3 project*).ti,ab,kf. |
| 324 | program development.ab,kf,ti. |
| 325 | or/303-324 |
| 326 | 167 and 285 and 302 and 325 |

# Ovid Embase search string

2023 November 1

| **#** | **Query** |
| --- | --- |
| 1 | "scandinavian and nordic countries"/ or denmark/ or greenland/ or norway/ or svalbard/ or sweden/ or finland/ or iceland/ |
| 2 | (denmark or danmark or sweden or sverige or norway or norge or finland or iceland).ab,in,kf,ti. |
| 3 | (denmark* or dane* or danish or sweden* or swede* or swedish or norway* or norwegian* or finland or finn* or iceland or icelandic* or greenland or greenlandic* or scandinavia* or nordic*).ab,kf,ti. |
| 4 | or/1-3 |
| 5 | exp exercise/ |
| 6 | exp health education/ |
| 7 | exp physiotherapy/ |
| 8 | exp rehabilitation/ |
| 9 | motor activity/ |
| 10 | social behavior/ |
| 11 | exp motivation/ |
| 12 | sedentary lifestyle/ |
| 13 | exp self control/ |
| 14 | exp kinesiotherapy/ |
| 15 | occupational therapy/ |
| 16 | recreational therapy/ |
| 17 | convalescence/ |
| 18 | "exercise*".ab,kf,ti. |
| 19 | fitness.ab,kf,ti. |
| 20 | physical effort.ab,kf,ti. |
| 21 | physical exertion.ab,kf,ti. |
| 22 | athletic performance.ab,kf,ti. |
| 23 | calisthenics.ab,kf,ti. |
| 24 | exergaming.ab,kf,ti. |
| 25 | "gymnastic*".ab,kf,ti. |
| 26 | cool down.ab,kf,ti. |
| 27 | pilates.ab,kf,ti. |
| 28 | "plyometric*".ab,kf,ti. |
| 29 | squatting.ab,kf,ti. |
| 30 | warm up.ab,kf,ti. |
| 31 | health education.ab,kf,ti. |
| 32 | "health fair*".ab,kf,ti. |
| 33 | (education and (breast feeding or childbirth or death or dental health or diabetes or hiv or nutrition or parenting or patient or preoperative or psycho or school health or self care or sleep)).ab,kf,ti. |
| 34 | health literacy.ab,kf,ti. |
| 35 | health promotion.ab,kf,ti. |
| 36 | (Physiotherapies or physiotherapy).ab,kf,ti. |
| 37 | physical therapy.ab,kf,ti. |
| 38 | chest wall oscillation*.ab,kf,ti. |
| 39 | (joint mobilization* or joint mobilisation*).ab,kf,ti. |
| 40 | kinesio taping.ab,kf,ti. |
| 41 | (resocialisation or resocialization or revalidation).ab,kf,ti. |
| 42 | bibliotherap*.ab,kf,ti. |
| 43 | community reintegration.ab,kf,ti. |
| 44 | constraint induced therap*.ab,kf,ti. |
| 45 | drug dependence treatment*.ab,kf,ti. |
| 46 | functional assessment*.ab,kf,ti. |
| 47 | mental health recovery.ab,kf,ti. |
| 48 | "mirror therap*".ab,kf,ti. |
| 49 | (occupational therapies or occupational therapy).ab,kf,ti. |
| 50 | (recreational therapy or recreational therapies).ab,kf,ti. |
| 51 | "sociotherap*".ab,kf,ti. |
| 52 | sensorimotor integration.ab,kf,ti. |
| 53 | (patient* adj5 "self report").ab,kf,ti. |
| 54 | motor activit*.ab,kf,ti. |
| 55 | (social behavior or social behaviour).ab,kf,ti. |
| 56 | motivation.ab,kf,ti. |
| 57 | aspirational.ab,kf,ti. |
| 58 | (incentive adj5 behavio*).ab,kf,ti. |
| 59 | sedentary lifestyle.ab,kf,ti. |
| 60 | self control.ab,kf,ti. |
| 61 | "emotion* regulation".ab,kf,ti. |
| 62 | "kinesiotherap*".ab,kf,ti. |
| 63 | "kinesitherap*".ab,kf,ti. |
| 64 | neuromuscular facilitation.ab,kf,ti. |
| 65 | (tai chi or tai ji).ab,kf,ti. |
| 66 | yoga.ab,kf,ti. |
| 67 | convalescence.ab,kf,ti. |
| 68 | ((physical adj3 workout) or (physical adj3 work-out)).ab,kf,ti. |
| 69 | "activit* of daily living ".ab,kf,ti. |
| 70 | "recovery of function".ab,kf,ti. |
| 71 | "leisure activit*".ab,kf,ti. |
| 72 | "run*".ab,kf,ti. |
| 73 | "jog*".ab,kf,ti. |
| 74 | "swim*".ab,kf,ti. |
| 75 | "walk*".ab,kf,ti. |
| 76 | stair climbing.ab,kf,ti. |
| 77 | (blood flow restriction training or blood flow restriction therap*).ab,kf,ti. |
| 78 | (movement therap* or motion therap*).ab,kf,ti. |
| 79 | sex education.ab,kf,ti. |
| 80 | smoking prevention.ab,kf,ti. |
| 81 | healthy people program*.ab,kf,ti. |
| 82 | prenatal education.ab,kf,ti. |
| 83 | "animal assisted therap*".ab,kf,ti. |
| 84 | "equine assisted therap*".ab,kf,ti. |
| 85 | Dry needling.ab,kf,ti. |
| 86 | "electric stimulation therap*".ab,kf,ti. |
| 87 | electroacupuncture.mp. or electro acupuncture.ab,kf,ti. |
| 88 | pulsed radiofrequency treatment.ab,kf,ti. |
| 89 | spinal cord stimulation.ab,kf,ti. |
| 90 | Qigong.ab,kf,ti. |
| 91 | "extracorporeal shockwave therap*".ab,kf,ti. |
| 92 | "hydrotherap*".ab,kf,ti. |
| 93 | "aquatic therap*".ab,kf,ti. |
| 94 | "therap* soft tissue".ab,kf,ti. |
| 95 | massage.ab,kf,ti. |
| 96 | manual lymphatic drainage.ab,kf,ti. |
| 97 | "art therap*".ab,kf,ti. |
| 98 | early ambulation.ab,kf,ti. |
| 99 | (cooperative behavior or cooperative behaviour).ab,kf,ti. |
| 100 | empowerment.ab,kf,ti. |
| 101 | "achievement*".ab,kf,ti. |
| 102 | (sedentary behavior or sedentary behaviour).ab,kf,ti. |
| 103 | (sport* adj3 rehab*).ab,kf,ti. |
| 104 | "recreation*".ab,kf,ti. |
| 105 | dancing.ab,kf,ti. |
| 106 | "hobb*".ab,kf,ti. |
| 107 | horticulture.ab,kf,ti. |
| 108 | gardening.ab,kf,ti. |
| 109 | hunting.ab,kf,ti. |
| 110 | "game*".ab,kf,ti. |
| 111 | physical endurance.ab,kf,ti. |
| 112 | (baseball or basketball or bicycl* or boxing or cricket or football or golf or hockey or martial art* or mountaineering or tennis or rugby or skating or skiing or soccer or "track and field" or volleyball or diving or "weight lifting" or wrestling).ab,kf,ti. |
| 113 | (health behavior* or health behaviour*).ab,kf,ti. |
| 114 | (health risk behavior* or health risk behaviour*).ab,kf,ti. |
| 115 | "rehabilit*".ab,kf,ti. |
| 116 | "training*".ab,kf,ti. |
| 117 | "sprint*".ab,kf,ti. |
| 118 | or/5-117 |
| 119 | integrated health care system/ |
| 120 | clinical handover/ |
| 121 | exp hospital discharge/ |
| 122 | integrated health care system.ab,kf,ti. |
| 123 | (integrat* adj3 care).ab,kf,ti. |
| 124 | (patient* handoff or patient* hand off or patient* transition).ab,kf,ti. |
| 125 | "inter sector* or intersector*".ab,kf,ti. |
| 126 | (multi sector* or multisector* or multi-sector*).ab,kf,ti. |
| 127 | (continue* adj3 care).ab,kf,ti. |
| 128 | (discharg* adj2 (home or hospital* or center* or centre* or clinic*)).ab,kf,ti. |
| 129 | clinical handover.ab,kf,ti. |
| 130 | patient care/ |
| 131 | (patient* adj3 transfer*).ab,kf,ti. |
| 132 | (cross sector* or cross-sector* or across sector* or a cross sector* or between sector*).ab,kf,ti. |
| 133 | (patient* adj3 trajector*).ab,kf,ti. |
| 134 | (patient* adj3 pathway*).ab,kf,ti. |
| 135 | delivery of health care.ab,kf,ti. |
| 136 | or/119-135 |
| 137 | exp implementation science/ |
| 138 | translational research/ |
| 139 | health care planning/ |
| 140 | health plan implementation.ab,kf,ti. |
| 141 | health planning.ab,kf,ti. |
| 142 | health priorities.ab,kf,ti. |
| 143 | health resource*.ab,ti,kf. |
| 144 | health impact assessment/ |
| 145 | health impact assessment*.ab,kf,ti. |
| 146 | social aspect/ |
| 147 | social planning.ab,kf,ti. |
| 148 | total quality management/ |
| 149 | quality improvement*.ab,kf,ti. |
| 150 | program development/ |
| 151 | health care quality/ |
| 152 | quality of health care.ab,kf,ti. |
| 153 | outcome assessment/ |
| 154 | (outcome assessment* or process assessment*).ab,kf,ti. |
| 155 | quality control/ |
| 156 | quality control.ab,kf,ti. |
| 157 | quality assurance.ab,kf,ti. |
| 158 | "implementation science*".ab,kf,ti. |
| 159 | translational research.ab,kf,ti. |
| 160 | total quality management.ab,kf,ti. |
| 161 | implement*.ab,kf,ti. |
| 162 | (evaluat* adj3 project*).ab,kf,ti. |
| 163 | (evaluat* adj3 (programme or program or programs)).ab,kf,ti. |
| 164 | or/137-163 |
| 165 | 4 and 118 and 136 and 164 |

# CINAHL (EBSCO host) search string

2023 November 1

|  |  |
| --- | --- |

| S120 | S6 AND S91 AND S103 AND S119 |  |
| --- | --- | --- |
| S119 | S104 OR S105 OR S106 OR S107 OR S108 OR S109 OR S110 OR S111 OR S112 OR S113 OR S114 OR S115 OR S116 OR S117 OR S118 |  |
| S118 | Program development |  |
| S117 | implement* |  |
| S116 | ((evaluat*) N3 (project*)). |  |
| S115 | outcome assessment or process assessment |  |
| S114 | ((evaluat*) N3 (programme or program or programs)). |  |
| S113 | quality of health care or quality assessment or quality improvement |  |
| S112 | (MH "Quality of Health Care") OR (MH "Quality Assessment") OR (MH "Quality Improvement") |  |
| S111 | social planning |  |
| S110 | resource-limited setting* |  |
| S109 | health impact assessment |  |
| S108 | health care reform or health priorit* or health resource allocation or health resource utilization |  |
| S107 | (MH "Health Care Reform") OR (MH "Health Priorities") OR (MH "Health Resource Allocation") OR (MH "Health Resource Utilization") |  |
| S106 | health planning |  |
| S105 | (MH "Translational Medical Research") OR "translational research" |  |
| S104 | (MH "Implementation Science") |  |
| S103 | S92 OR S93 OR S94 OR S95 OR S96 OR S97 OR S98 OR S99 OR S100 OR S101 OR S102 |  |
| S102 | (discharg* N3 (home or hospital* or center* or centre* or clinic*)) |  |
| S101 | (MH "Transfer, Discharge") |  |
| S100 | (patient* N3 transfer) or (patient* N3 pathway*) or (patient* N3 trajector*) |  |
| S99 | (multi sector* or multisector* or multi-sector*) |  |
| S98 | ((cross sector* or across sector* or a cross sector* or between sector*) |  |
| S97 | inter sector* or intersector* |  |
| S96 | (intergrat* N3 care) |  |
| S95 | patient hand off or patient handoff or patient transition* or clinical handover |  |
| S94 | (continuit* N3 care) |  |
| S93 | (MH "Health Care Delivery, Integrated") |  |
| S92 | (MH "Continuity of Patient Care+") |  |
| S91 | S7 OR S8 OR S9 OR S10 OR S11 OR S12 OR S13 OR S14 OR S15 OR S16 OR S17 OR S18 OR S19 OR S20 OR S21 OR S22 OR S23 OR S24 OR S25 OR S26 OR S27 OR S28 OR S29 OR S30 OR S31 OR S32 OR S33 OR S34 OR S35 OR S36 OR S37 OR S38 OR S39 OR S40 OR S41 OR S42 OR S43 OR S44 OR S45 OR S46 OR S47 OR S48 OR S49 OR S50 OR S51 OR S52 OR S53 OR S54 OR S55 OR S56 OR S57 OR S58 OR S59 OR S60 OR S61 OR S62 OR S63 OR S64 OR S65 OR S66 OR S67 OR S68 OR S69 OR S70 OR S71 OR S72 OR S73 OR S74 OR S75 OR S76 OR S77 OR S78 OR S79 OR S80 OR S81 OR S82 OR S83 OR S84 OR S85 OR S86 OR S87 OR S88 OR S89 OR S90 |  |
| S90 | rehab* N5 care |  |
| S89 | training* |  |
| S88 | rehabilit* |  |
| S87 | physical workout or physical work-out |  |
| S86 | convalescence |  |
| S85 | kinesitherap* |  |
| S84 | kinesiotherap* |  |
| S83 | (incentive N5 behavio*) |  |
| S82 | (patient* N5 "self report") |  |
| S81 | sensorimotor integration |  |
| S80 | "sociotherap*" |  |
| S79 | mirror therap* |  |
| S78 | mental health recovery |  |
| S77 | functional assessment* |  |
| S76 | drug dependence treatment* |  |
| S75 | constraint induced therap* |  |
| S74 | community reintegration |  |
| S73 | (MH "Community Reintegration") |  |
| S72 | (resocialisation or resocialization or revalidation) |  |
| S71 | joint mobilization or joint mobilisation or kinesio taping |  |
| S70 | chest wall oscillation* |  |
| S69 | cool down or warm up or plyometric* or pilates or squatting |  |
| S68 | calisthenics |  |
| S67 | physical exertion |  |
| S66 | physical effort* |  |
| S65 | kinesiotherap* |  |
| S64 | (baseball or basketball or bicycl* or boxing or cricket or football or golf or hockey or "martial arts" or mountaineering or tennis or rugby or skating or skiing or soccer or "track and field" or volleyball or diving or "weight lifting" or wrestling) |  |
| S63 | athletic performance or fitness or physical endurance |  |
| S62 | game* |  |
| S61 | horticulture or gardening or hunting |  |
| S60 | dancing or hobb* |  |
| S59 | recreation* |  |
| S58 | sport* N3 rehab* |  |
| S57 | emotion* regulation |  |
| S56 | sedentary behavior or sedentary behaviour |  |
| S55 | self control |  |
| S54 | achievement* or aspirational |  |
| S53 | empowerment |  |
| S52 | cooperative behavior or cooperative behaviour |  |
| S51 | early ambulation |  |
| S50 | (MH "Early Ambulation") |  |
| S49 | art therap* or bibliotherap* |  |
| S48 | manual lymphatic drainage |  |
| S47 | massage |  |
| S46 | hydrotherap* or aquatic therap* or soft tissue therap* |  |
| S45 | extracorporeal shockwave therap* |  |
| S44 | motion therap* or movement therap* |  |
| S43 | blood flow restriction training or blood flow restriction therap* |  |
| S42 | tai ji or tai chi |  |
| S41 | yoga |  |
| S40 | qigong |  |
| S39 | spinal cord stimulation |  |
| S38 | pulsed radiofrequency treatment* |  |
| S37 | dry needling or electric stimulation therap* or electroacupunture or eletro acupuncture |  |
| S36 | animal assisted therap* or equine assisted therap* |  |
| S35 | physical therapy modalit* |  |
| S34 | prenatal education |  |
| S33 | health literacy |  |
| S32 | smoking prevention |  |
| S31 | (education and (breast feeding or childbirth or death or dental health or diabetes or hiv or nutrition or parenting or patient or preoperative or psycho or school health or self care or sleep)) |  |
| S30 | health fair* or health promotion or sex education |  |
| S29 | (MH "Health Fairs") |  |
| S28 | run* or swim* or walk* or jog* or sprint* or stair climbing |  |
| S27 | gymnastic* |  |
| S26 | exergaming |  |
| S25 | exercis* or health education or physical therapy or physical therapies occupational therapy or occupational therapies |  |
| S24 | health behavior og health behaviour or health risk behavior or health risk behaviour |  |
| S23 | (MH "Health Behavior") |  |
| S22 | leisure activit* |  |
| S21 | recovery |  |
| S20 | motor activit* or motivation or sedentary lifestyle or social behavior or social behaviour or self regulation or recreational therap* |  |
| S19 | (MH "Leisure Activities+") |  |
| S18 | physical* N3 activit* |  |
| S17 | activit* of daily living |  |
| S16 | (MH "Recovery+") |  |
| S15 | (MH "Recreational Therapy") |  |
| S14 | (MH "Self Regulation+") |  |
| S13 | (MH "Social Behavior") |  |
| S12 | (MH "Life Style, Sedentary+") |  |
| S11 | (MH "Motivation") |  |
| S10 | (MH "Motor Activity+") |  |
| S9 | (MH "Physical Therapy+") OR (MH "Occupational Therapy+") OR (MH "Home Rehabilitation+") OR (MH "Rehabilitation+") |  |
| S8 | (MH "Health Education+") |  |
| S7 | (MH "Exercise+") |  |
| S6 | S1 OR S2 OR S3 OR S4 OR S5 |  |
| S5 | AF ((denmark or danmark or sweden or sverige or norway or norge or finland or iceland)) |  |
| S4 | (denmark* or dane* or danish or sweden* or swede* or swedish or norway* or norwegian* or finland or finn* or iceland or icelandic* or greenland or greenlandic* or scandinavia* or nordic*) |  |
| S3 | (MH "Scandinavia+") |  |
| S2 | (MH "Denmark") OR (MH "Greenland") OR (MH "Sweden") OR (MH "Norway") OR (MH "Finland") OR (MH "Iceland") |  |
| S1 | (MH "Scandinavians and Nordic Persons") |  |

# PEDro, OTseeker, Klinisk sygepleje (via Idun)

2023 November 1

**PEDro**

| **Søgning** |
| --- |
| Rehabilitation AND sector* AND implementation |
| Rehabilitation AND sector* AND evaluation |

**OTseeker**

| **Søgning** |
| --- |
| Rehabilitation AND sector* AND implementation |
| Rehabilitation AND sector* AND evaluation |

**Klinisk Sygepleje**

| **Search** |
| --- |
| rehabilitation  AND  sector* OR cross OR across OR a cross OR between OR cross-sector*  AND  implementation OR evaluation    Published in: Klinisk Sygepleje |
| rehabilitering  AND  sektor OR tværgående OR på tværs OR mellem OR tværsektoriel  AND  implementering OR evaluering    Published in: Klinisk Sygepleje |
| rehabilitering  AND  sektor OR tvärgående OR tvärs OR mellan OR emellan OR tvärsektoriellt  AND  implementering OR genomföande OR genomföring OR utvärdering    Published in: Klinisk Sygepleje |
| rehabilitering  AND  sektor OR tverrsektoriell OR tverrgående  AND  implementering OR evaluering OR gjennomføring    Published in: Klinisk Sygepleje |
